# Factors associated with severe maternal outcomes in patients with eclampsia in an obstetric intensive care unit: A cohort study

**DOI:** 10.1097/MD.0000000000027313

**Published:** 2021-09-24

**Authors:** Joanna Francyne Silva De Barros, Melania Maria Amorim, Duana Gabrielle De Lemos Costa, Leila Katz

**Affiliations:** a Instituto de Medicina Integral Professor Fernando Figueira, Recife, Pernambuco, Brazil; b Stricto Sensu Postgraduate Program, Instituto de Medicina Integral Prof. Fernando Figueira , Recife, Pernambuco, Brazil.

**Keywords:** eclampsia, hypertensive disorders, maternal death, maternal near-miss, preeclampsia

## Abstract

To describe the clinical profile, management, maternal outcomes and factors associated with severe maternal outcome (SMO) in patients admitted for eclampsia.

A retrospective cohort study was carried out. All women admitted to the Obstetric Intensive Care Unit (ICU) at Instituto de Medicina Integral Prof. Fernando Figueira (IMIP), Recife, Northeast of Brazil, from April 2012 to December 2019 were considered for inclusion and patients with the diagnosis of eclampsia were selected. Patients who, after reviewing their medical records, did not present a diagnosis of eclampsia were excluded from the study. Severe maternal outcome (SMO) was defined as all cases of near miss maternal mortality (MNM) plus all maternal deaths during the study period. The Risk Ratio (RR) and its 95% confidence interval (95% CI) were calculated as a measure of the relative risk. Multiple logistic regression analysis was performed to control confounding variables. The institute's internal review board and the board waived the need of the informed consent.

Among 284 patients with eclampsia admitted during the study period, 67 were classified as SMO (23.6%), 63 of whom had MNM (22.2%) and 5 died (1.8%). In the bivariate analysis, the following factors were associated with SMO: age 19 years or less (RR = 0.57 95% CI 0.37–0.89, *P* = .012), age 35 years or more (RR = 199 95% CI 1.18–3.34, *P* = .019), the presence of associated complications such as acute kidney injury (RR = 3.85 95% CI 2.69–5.51, *P* < .001), HELLP syndrome (RR = 1.81 95% CI 1.20–2.75, *P* = .005), puerperal hemorrhage (PPH) (RR = 2.15 95% CI 1.36–3.40, *P* = .003) and acute pulmonary edema (RR = 2.78 95% CI 1.55–4.96, *P* = .008). After hierarchical multiple logistic regression analysis, the factors that persisted associated with SMO were age less than or equal to 19 years (ORa = 0.46) and having had PPH (ORa = 3.33).

Younger age was a protective factor for developing SMO, while those with PPH are more likely to have SMO.

## 1 Introduction

Around 810 women die daily worldwide from complications in pregnancy and/or childbirth.^[1]^ Hypertensive disorders, particularly eclampsia, remain the leading cause of maternal mortality in Brazil^[2,3]^ and account for 14% of global maternal mortality, corresponding to 30,000 deaths annually.^[4]^

Eclampsia is characterized by tonic-clonic seizures of early onset in the absence of epilepsy, ischemia, intracranial hemorrhage or drug use.^[5]^ The disease is multifactorial and preventive actions should address lifestyle, diet and family and/or obstetric history.^[6–8]^

Most hypertension-related maternal deaths occur intrapartum or in the immediate postpartum due to avoidable and treatable causes.^[4]^ Therefore, the survival of women with eclampsia could serve as an indicator of the quality of care and its determinants.^[9]^ Because these deaths are relatively rare, the World Health Organization (WHO) developed the concept of maternal near-miss (MNM), defined as the presence of organ failure or organ dysfunction during pregnancy, childbirth or postpartum.^[9]^

Another relevant concept is severe maternal outcome (SMO), defined as cases of death plus MNM.^[9]^ Although the prevalence of SMO is five-fold in eclampsia compared to other hypertensive disorders, few such studies have been conducted in Brazil.^[10]^ Therefore, this study aimed to describe the clinical profile, management, maternal outcomes and factors associated with SMO in patients with eclampsia admitted to an obstetric intensive care unit (ICU) in Northeastern Brazil.

## 2 Methods

This retrospective cohort study was conducted between March 2017 and December 2019 in the obstetric ICU of the *Instituto de Medicina Integral Prof. Fernando Figueira* (IMIP), a tertiary referral hospital in Recife, Pernambuco, Brazil. The institute's internal review board approved the protocol under reference CAAE 84895318.6.00005201, and the board waived the need of the informed consent. STROBE recommendations for reporting observational studies were followed.

Between April 2012 and December 2019, 388 women were admitted to the obstetric ICU with eclampsia. Women who died at admission and those whose records could not be found were excluded.

Eclampsia was defined as the occurrence of focal or multifocal tonic-clonic seizures in the absence of other clinical conditions such as epilepsy, ischemia and intracranial hemorrhage, or drug use.^[5]^ Treatment consisted of magnesium sulfate (an intravenous loading dose of 6 grams of MgSO_4_ and an intravenous maintenance dose of 2 grams/hour for 24 hours after the last seizure), oxygen therapy and maintenance of patent airways. Following stabilization, the pregnancy was interrupted, although neither immediately nor inopportunely, but rather when the condition was under control and laboratory parameters, particularly platelet count, are available. Induction was offered at gestational age ≥34 weeks, or before that in the case of fetal death or a non-viable pregnancy. Eclampsia is not considered per se an indication for cesarean delivery; however, the earlier the gestational age and the more unfavorable the cervical conditions, the more likely the procedure is.^[11]^

The independent predictive variables were: age, ethnicity/skin color, number of pregnancies and deliveries, years of schooling, place of residence, seizures at home/ before hospitalization, antepartum eclampsia, gestational age at eclampsia and delivery, mode of delivery, chronic hypertension, acute kidney injury (AKI), HELLP syndrome, postpartum hemorrhage (PPH), acute pulmonary edema (APE), number of convulsive seizures, recurrence of eclampsia, inadequate MgSO_4_ regimen (inappropriate dose/duration, discontinuation, or use of another anticonvulsant instead of MgSO_4_), blood transfusion, assisted mechanical ventilation (AMV), and additional anticonvulsants.

SMO was the dependent variable. All deaths occurring during pregnancy, childbirth and up to 42 days postpartum were taken into consideration^[5]^ and the WHO criteria for MNM were adopted.^[9]^

Epi Info, version 3.5.4 (Atlanta, GA, USA) and Medcalc 19.8 (Medcalc Software Ltda) were used to create a database and to perform the statistical analysis. Frequency distribution tables were used to calculate measures of central tendency and dispersion. Student *t* test was used for two-group comparison of continuous variables with normal distribution and the Mann–Whitney test for non-normal distributions and discrete or ordinal variables. The categorical variables were compared in contingency tables using Pearson's Chi-Squared test of association and Fisher exact test, as appropriate.

Risk ratios and their 95% confidence intervals (95% CI) were calculated to determine the strength of the association between the independent predictive variables and SMO. A 5% significance level was adopted, and all *P* values were two-tailed.

In a hierarchical logistic regression analysis, the variables were entered according to levels. In each block, the variables that remained associated with the outcome at a significance level of 20% were selected and analyzed again until the only variables that remained were those associated with the endpoint at a significance level of 5%. These were then included in a new regression analysis to calculate adjusted risk estimates. A hierarchical multiple logistic regression analysis was then performed to control for confounding variables. Different models were tested. Since AKI, HELLP, and AMV are criteria for MNM, and AMV is also a marker of greater severity, these factors were removed from the final analysis.

## 3 Results

**Figure 1:**
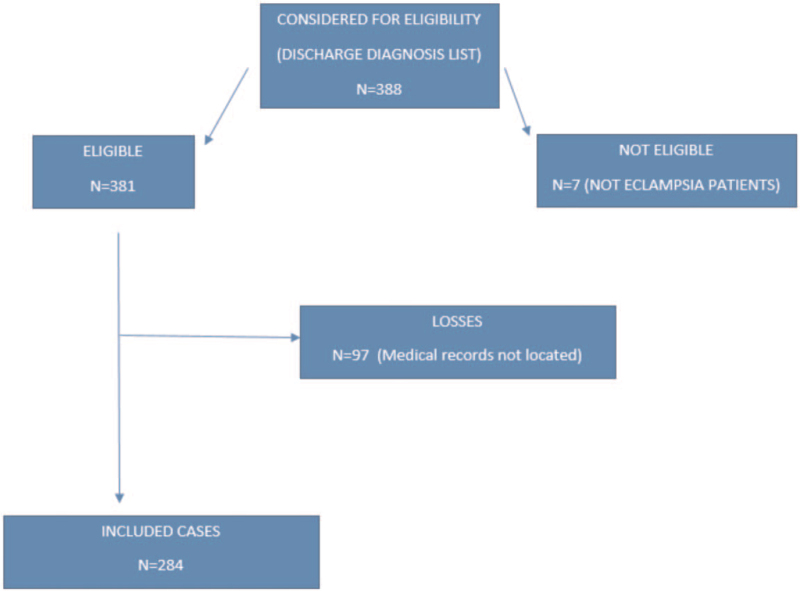
Flow diagram of participants.

During the study period, 388 women were admitted to IMIP's obstetric ICU with eclampsia. Of these, 97 had incomplete/irretrievable records, and in 7, diagnosis was unconfirmed, leaving a total of 284 participants. There were 63 cases of MNM (22.2%) and 5 deaths (1.8%), resulting in a frequency of SMO of 23.9% (n = 68) (Fig. 1).

SMO increased with age (*P* = .007). Patients ≤19 years (37.3%) had a 37% lower risk of SMO (*P* = .012), whereas those ≥35 years had a two-fold greater risk (*P* = .019). Obstetric history was similar in the overall sample. Of the participants, 79.8% were brown-skinned, 10.1% white, 9.6% black, and only 1 woman was indigenous. No association was found between an SMO and ethnicity/skin color (data available in 178 patients), years of schooling (data available in 117 patients), place of residence (the state capital/another town), or how the patient had arrived at the institute (from home/hospital transfer) (Table 1).

**Table 1:** Clinical and sociodemographic characteristics of the patients with eclampsia.

	Adverse maternal outcome				
Maternal characteristics	Yes (n = 68)	No (n = 217)	RR	95% CI	*P* value
Age, y (mean ± SD)	24.3 (8.1)	21.1 (6.7)	–	–	.001
Age <19 years (n/%)	25 (37.3)	119 (54.8)	0.57	0.37–0.89	.012
Age ≥35 y (n/%)	10 (14.9)	13 (6.0)	1.99	1.18–3.34	.002
Ethnicity/skin color (brown or black)a	33 (91.7)	127 (89.4)	1.23	0.42–3.63	.69
Number of pregnancies (median /IQR)	1 (1–2)	1 (1–2)	–	–	.14^∗^
Parity (median/IQR)	0.5 (0–2)	0 (0–1)	–	–	.10^∗^
Primigravida (n/%)	38 (59.4)	149 (69.3)	0.71	0.46–1.10	.14
Multiparous (n/%)	17 (26.6)	41 (19.1)	1.37	0.85–2.21	.19
<8 y of schooling (n/%) b	12 (63.2)	52 (53.1)	1.41	0.60–3.34	.41
Came directly from home to hospital (n/%)	3 (4.5)	10 (4.6)	0.97	0.35–2.69	.96
Seizures at home (n/%)	10 (15.2)	36 (16.7)	0.91	0.50–1.65	.76
Lives outside the state capital (n/%)	41 (61.2)	118 (54.4)	1.24	0.80–1.90	.32
Eclampsia prior to hospital admission (n/%)	55 (82.1)	180 (83.7)	0.91	0.53–1.57	.75
Eclampsia prior to delivery (n/%)	47 (70.1)	157 (72.4)	0.92	0.58–1.45	.72
Gestational age at eclampsia (mean ± SD) c	33.6 (5.4)	34.5 (4.2)	–	–	.56
Gestational age ≤34 weeks at delivery (n/%)	22 (47.8)	68 (43.3)	1.15	0.69–1.91	.58
Cesarean section (n/%)	53 (79.1)	175 (80.6)	0.92	0.55–1.55	.78
Chronic arterial hypertension (n/%)	16 (23.9)	59 (27.2)	0.87	0.53–1.43	.59
Acute kidney injury (n/%)	25 (37.3)	13 (6.0)	3.85	2.69–5.51	<.001
HELLP (n/%)	27 (40.3)	50 (23.0)	1.81	1.20–2.75	.005
Hemorrhage (n/%)	14 (20.9)	17 (7.8)	2.15	1.36–3.40	.003
Acute pulmonary edema (n/%)	5 (7.5)	3 (1.4)	2.78	1.55–4.96	.008^†^
Number of seizures (median/IQR)	2 (1–2.5)	2 (1–2)	–	–	.76^∗^
≥2 seizures (n/%)	27 (51.9)	95 (50.3)	1.05	0.65–1.70	.83
Recurrence (n/%)	8 (11.9)	31 (14.4)	0.84	0.43–1.72	.60
Inappropriate regimen at IMIP (n/%)	3 (4.5)	11 (5.1)	0.90	0.32–0.84	.57^†^
Blood transfusion (n/%)	7 (10.4)	14 (6.5)	1.46	0.76–2.78	.20^†^
Assisted mechanical ventilation (n/%)	34 (50.7)	13 (6.0)	5.19	3.61–7.46	<.001
Other anticonvulsants (n/%)	11 (17.5)	27 (15.9)	1.08	0.72–1.88	.77

IMIP = Instituto de Medicina Integral Prof. Fernando Figueira, IQR = interquartile range.

∗ Mann–Whitney test.

† Fisher's exact test; ^a^Data available for 178 patients; ^b^Data available for 117 patients; c Data available for 188 patients (the remainder were admitted postpartum).

Seizures occurred before delivery in 72.6% of the patients, postpartum in 25.6%, and during delivery in 1.8%. The occurrence of SMO did not vary with the time at which eclampsia occurred in relation to hospitalization and delivery. Mean gestational age at eclampsia was 34 weeks in women with an SMO and 34 weeks and 5 days in those without (*P* = .56). The Cesarean section rate was 79.1%; however, the risk of an SMO was no higher with this delivery mode (Table 1).

Chronic hypertension was the most common comorbidity; however, it was not associated with SMO. AKI was more common in the women with an SMO (37.3%) compared to those without (6.0%) (RR = 3.85; 95% CI: 2.69–5.51; *P* < .001). HELLP syndrome was also a risk factor for SMO (40.3% vs 23.0%, respectively) (RR = 1.81; 95% CI: 1.20–2.75; *P* = .0050) as were PPH (20.9% vs 7.8%; RR = 2.15; 95% CI: 1.36–3.40; *P* = .003) and APE (7.5% vs 1.4%; RR = 2.78; 95% CI: 1.55–4.96; *P* = .008) (Table 1).

The median number of seizures was 2 (interquartile range [IQR] 1–2.5; *P* = .76), with the worst case being a woman who suffered 14 seizures. The risk of progressing to an SMO was no higher in women who had ≥2 seizures (51.9%).

Recurrent eclampsia at some moment was reported in 62 cases (21.8%), with 39 (13.8%) occurring within the institute. The frequency of SMO was similar between the women who had a recurrence at IMIP compared to those who did not (11.9% vs 14.4%; RR = 0.84; 95%CI: 0.43–1.72; *P* = .60) (Table 1).

MgSO_4_ was inadequately used at IMIP in 14 cases, with no difference between the SMO and non-SMO groups (4.5% vs 5.1%; RR = 0.90; 95% CI: 0.32–0.84; *P* = .56) (Table 1).

AMV was required in 50.7% of cases with SMO compared to 6.0% of those without (RR = 5.19; 95% CI: 3.61–7.46; *P* < .001) and blood transfusion was required in 10.4% vs 6.5%, respectively (RR = 1.46; 95% CI: 0.76–2.78; *P* = .20) (Table 1).

In the multiple logistic regression analysis, PPH (adjusted OR = 3.33; 95% CI: 1.52–7.33; *P* = .002) and age < 19 years (adjusted OR = 0.46; 95% CI: 0.26–0.82; *P* = .009) remained associated with an SMO (Table 2), this model predicted correctly 76.4% of cases.

**Table 2:** Conditions associated with an adverse maternal outcome, following multivariate analysis, in patients diagnosed with eclampsia.

Predictive factor	Coefficient	Standard error	Odds ratio	95% CI	*P* value
Postpartum hemorrhage	1.20	0.40	3.33	1.52–7.33	.002
Age <19 y	−0.76	0.29	0.46	0.26–0.82	.009
Constant	−0.9860	0.1939	^∗^	^∗^	.0000

IMIP = Instituto de Medicina Integral Prof. Fernando Figueira. Area under the ROC curve (AUC): 0.64 standard error: 0.03 95% confidence interval: 0.58–0.70.

## 4 Discussion

Eclampsia was an important cause of SMO in this study, with associated factors being maternal age <19 years (a protective factor) and concomitant PPH.

Despite all the advances, hypertensive syndromes, particularly eclampsia, remain a major cause of maternal mortality.^[2,9]^ Deaths from eclampsia vary according to the level of development in the region,^[12]^ ranging from no cases in a Finnish series^[8]^ to 21.3% in Nigeria.^[13]^ In poor countries, not only is the risk of preeclampsia greater but the risk of progression to eclampsia is also higher and the risk of maternal death is very high.^[12]^ Conversely, a higher human development index (HDI) is associated with lower rates of eclampsia and mortality.^[6,10,14,15]^ Several factors influence this variation, including the population size, event frequency, quality of life and quality of care, particularly prenatal and postpartum care.^[10,13,16–18]^

The death rate found here (1.8%) is relatively low considering the region evaluated, possibly because IMIP is a teaching institute and a referral hospital, with an experienced obstetric ICU staff and strict clinical protocols. Other teaching hospitals in developing countries have reported similar rates.^[19,20]^ Nonetheless, the frequent process of transferring critical patients from other hospitals/regions contributes towards increasing the mortality rate, which, although low, was not zero.

MNM, often associated with hypertensive disorders,^[21]^ particularly eclampsia, had a frequency of 22.2%, which is in agreement with previous reports (14%–34%).^[6,22,23]^ However, unlike the mortality rate, it tended towards the high end of this range. Referral services attract extremely severe cases, as corroborated by the high complication and recurrence rates despite the low rates of treatment failure. Since advanced support is provided, women recover, constituting cases of MNM, while maternal mortality remains low.

Recent studies have used the SMO approach, including deaths and cases of MNM.^[9]^ Studies have evaluated death and MNM from eclampsia separately; however, few studies have associated eclampsia alone with SMO.^[22]^ A multicenter Brazilian study on eclampsia and factors associated with SMO showed that maternal age >30 years increased the risk of severe complications while age ≤19 years reduced this risk,^[10]^ as found in the present bivariate analysis. In this study, age >35 years was associated with a risk of SMO, as corroborated by previous findings.^[21]^ In an observational study conducted in Pakistan, 32.9% of the women who died from eclampsia were ≥35 years of age, while being ≥25 years of age increased the risk of death 1.5-fold.^[24]^

The risk of death was lower in adolescents, even after controlling for confounding factors. Young age is a known risk factor for hypertensive disorders^[18]^ and a social risk factor for poor quality prenatal care, possibly representing a bias.^[19,25,26]^ Nevertheless, the organic reserve in younger individuals may ensure a better prognosis for adolescents with eclampsia, although larger samples are required to confirm this effect. Conversely, the multicenter Brazilian study conducted to evaluate outcomes in women with eclampsia found no association between age and SMO.^[10]^

In this study, skin color was not associated with a risk of SMO. A multicenter Brazilian study found that black and brown-skinned women had a lower risk of progressing to an SMO,^[25]^ which was unexpected, since ethnicity/skin color is habitually interpreted as a marker of socioeconomic conditions that may lead to unfavorable outcomes.^[26]^ Nevertheless, those authors^[25]^ believed that the proportion of white participants could have been artificially high because skin color was self-reported. Furthermore, as in the present study in which data on ethnicity/skin color were available for only 62.6% of the sample, collecting data retrospectively frequently involves missing information and biases, emphasizing the importance of correctly recording data on patient records.

The association between nulliparity and eclampsia is already known.^[24]^ Theories suggest that early immune exposure to partners favors this condition.^[27]^ Furthermore, a change of partner in a second pregnancy would expose the woman to a similar risk as that experienced by nulliparas.^[28,29]^ In line with the findings of the multicenter Brazilian study, no association was found between parity and SMO^[10]^; however, the observational study conducted in Pakistan reported a 1.8-fold increased risk of death from eclampsia in nulliparas.^[24]^

Data on schooling were available in only 117 cases. Possibly as a result of this bias, no difference in schooling was found between the groups. A cross-sectional study conducted in less economically developed countries showed that only 37.5% of patients diagnosed with eclampsia had had >9 years of schooling.^[6]^ Nevertheless, although this indicator is a risk factor for eclampsia, our findings corroborate a previous study that failed to identify poor schooling as a risk factor for SMO.^[10]^

Arriving at this referral hospital directly from home did not alter the likelihood of an SMO. Delays in seeking healthcare have been confirmed as a risk factor for maternal death.^[15]^ In this sample, however, few women came directly from home and sample size would probably have to be larger to adequately evaluate this. Likewise, a retrospective cohort study conducted in a reference hospital in Africa showed that only 8/74 women arrived directly from home. In that tertiary hospital too, patients usually arrive transferred from other healthcare centers, since most women initially seek healthcare facilities of lower complexity, the most accessible care, when symptoms begin.^[30]^

The time until accessing healthcare negatively affects hypertensive disorders. In a study conducted in Norway, nulliparas living more than 1 hour from obstetric units had a 50% greater likelihood of developing eclampsia or HELLP, and this risk was doubled when the woman was multiparous.^[31]^

There was no association between the place of residence and the risk of an SMO. Although we were unable to identify studies in which the hospital location was specifically evaluated, the fact that the woman came from another town within the state, where hospitals have fewer resources, may theoretically have worsened her prognosis. A retrospective study evaluating complications and deaths in women with eclampsia found that the time between the seizure and the woman's arrival at the tertiary center was associated with greater rates of complications and maternal death.^[14]^ Another cross-sectional, nationwide Nigerian study reported similar results. In addition to the finding that the mean interval between diagnosis and definitive intervention in women with hypertension in general (preeclampsia and eclampsia) was >4 hours in 25% of the women who died, living >5 km from a hospital constituted a risk factor for maternal death in these women.^[22]^ Again, this information was missing from many of the charts, constituting a possible bias.

Most of the patients who developed eclampsia did so prior to delivery (71.8%), a finding similar to that reported from another cross-sectional study conducted in 14 Latin American hospitals in which 67.6% of the women had eclampsia prior to delivery.^[17]^ In the present study, when eclampsia did not occur antepartum, the cases occurred predominantly postpartum, unlike in other studies such as that conducted in Turkey in which 10% to 40% of eclampsia cases occurred intrapartum.^[20,32]^ Nevertheless, no association was found between the moment of the seizure or whether eclampsia occurred prior to delivery and the risk of an SMO.

Most of the women had already been diagnosed with eclampsia before admission to this hospital, while in 47 cases (18%), the first seizure occurred following admission. This frequency is similar to that found in a Dutch study.^[16]^ Although this situation may suggest greater severity, the risk of an SMO was no higher (*P* = .76), possibly due to a sample size bias.

Mean gestational age at birth was 34 weeks in this study, similar to data from other developing countries (34–35 weeks)^[20,33]^ but differing from the mean of close to 37 weeks reported for high-income countries.^[16]^ As in another Brazilian study, there was no difference in gestational age or in the rate of prematurity in relation to SMO.^[10]^ Nevertheless, a multicenter cross-sectional Brazilian study reported conflicting results.^[34]^

Although there is some controversy, the recommended type of delivery in cases of eclampsia is vaginal delivery, with induction of labor when possible.^[35]^ Nevertheless, Cesarean section rates were high in this study, as also reported previously.^[17,36]^ No association was found between mode of delivery and SMO, with similar C-section rates in both groups.

In a study in Latin American maternity hospitals, C-sections were associated with an increased risk of SMO even after controlling for confounding variables.^[21]^ In a Brazilian study, C-section was not associated with a risk of SMO in women with hypertensive disorders in general.^[34]^ A randomized clinical trial conducted in a tertiary hospital in India reported similar findings, with maternal outcome being slightly, although not significantly, better with C-sections.^[35]^

The frequency of chronic hypertension was quite high (26.4%); nevertheless, no association was found with SMO. A similar percentage was found in a tertiary center in Egypt,^[19]^ however, in a Dutch study, only 4% to 6% of the women with eclampsia had the condition^[18]^ and in a Finnish study with 46 cases of eclampsia only 2% had hypertension.^[8]^ These conflicting findings could be explained by socioeconomic and healthcare differences.

AKI was fairly common in this sample (13.3%). A cross-sectional study involving 29 countries of Africa, Asia, Latin America and the Middle East concluded that kidney failure was the third most common complication in eclampsia (19%), following neurological (52.4%) and respiratory dysfunction (33.3%).^[6]^ The association between AKI and SMO was unsurprising, since elevated creatinine levels (>3.5 mg/dL) and dialysis due to AKI constitute criteria for MNM.

HELLP syndrome was associated with a 4-fold increased risk of SMO in the present study. A Colombian study reported similar findings, with an increased risk of death for women with HELLP syndrome and eclampsia.^[36]^ Likewise, a retrospective Finnish study identified HELLP syndrome as the most common complication (22% of cases of eclampsia), despite the country's high HDI.^[8]^

APE was rare in this sample, occurring in only 7 women. Although the risk of SMO with this complication is high, no reports were found in the literature of an association between APE and SMO. However, this complication was also rare (1.8%) in cases of MNM in a high-risk maternity hospital of Northeastern Brazil.^[37]^

PPH, as a complication of eclampsia, was one of the main predictors of SMO in the present study, remaining significantly associated after controlling for confounding factors. Although the WHO defines PPH and eclampsia as potentially life-threatening conditions, a literature search failed to reveal any recent studies associating PPH with SMO in patients with eclampsia.^[38]^

In this study, SMO occurred in 4.5% of women exposed to the incorrect use of MgSO_4_ compared to 5.1% in those who were not. Nevertheless, the sample was small. The appropriate use of the anticonvulsant is crucial, not only in preventing the initial seizure but also in avoiding recurrences, a sign of clinical severity in a patient with eclampsia. In this study, 13.8% of the women had recurrent eclampsia; however, recurrence was not associated with SMO (*P* = .60). In some cases, repeat seizures occurred before the patient arrived at the institute, making confirmation impossible. Consequently, the true rate of recurrence may be higher. Nevertheless, the recurrence rate found here was higher than rates classically reported of around 9% to 10%^[39]^; however, it is probable that more severely ill patients with neurological complications are referred to this tertiary hospital, thus artificially increasing this rate. As previously shown, the presence of seizures depends on socioeconomic factors and can be aggravated by inappropriate care, increasing the risk of SMO five-fold.^[10,40]^

Treatment failure may result from the inappropriate use of MgSO_4_. Other anticonvulsants can be used if MgSO_4_ is unavailable or as follow-up treatment as clinically required. In this study, no association was found between the use of other anticonvulsants and SMO (around 17.5% in both groups); however, data were missing, particularly on treatment provided before the woman's admission to this hospital. It was impossible to compare these results with others, since this variable is not usually described or associated specifically with SMO.

Although common, blood transfusions were not associated with a risk of SMO in this study. A prospective multicenter African study showed that 98/288 women diagnosed with eclampsia received sufficient blood derivatives to fulfill the near-miss criteria; however, the association between this procedure and SMO was not analyzed.^[23]^ Blood transfusion is common in eclampsia, since high blood pressure can lead to greater hemorrhagic complications, particularly when HELLP syndrome is also present.

The frequency of AMV was significantly higher in women with SMO; however, AMV is not considered a cause or triggering factor of an SMO, but rather a marker of a more severe situation.

The factors that remained associated with SVO were maternal age <19 years and PPH, with age being a protective factor. Although hemorrhagic disorders in pregnancy are more common in women ≥35 years of age, this age range was not identified as a risk factor for SMO following regression analysis.^[41]^

A principal limitation of this study refers to its retrospective nature, since the data were obtained from patient charts from which data were often missing, mostly because the patient was referred from another facility or because of the woman's impaired state of consciousness at admission. The strengths of the study include its large sample size, achieved because this is an important referral center.

This study supports the need for healthcare services to create and implement strategies for improving reproductive healthcare planning, providing appropriate initial care at the original healthcare service, improving patient transfer, and providing timely and systematic care in tertiary services as a means of reducing SMO.

## Author contributions

**Conceptualization:** Joanna Francyne Silva De Barros, Melania Maria Amorim, Leila Katz.

**Data curation:** Joanna Francyne Silva De Barros, Melania Maria Amorim, Duana Gabrielle De Lemos Costa.

**Formal analysis:** Joanna Francyne Silva De Barros, Melania Maria Amorim, Duana Gabrielle De Lemos Costa, Leila Katz.

**Funding acquisition:** Joanna Francyne Silva De Barros, Duana Gabrielle De Lemos Costa.

**Investigation:** Joanna Francyne Silva De Barros, Duana Gabrielle De Lemos Costa, Leila Katz.

**Methodology:** Joanna Francyne Silva De Barros, Melania Maria Amorim, Duana Gabrielle De Lemos Costa, Leila Katz.

**Project administration:** Melania Maria Amorim, Leila Katz.

**Resources:** Joanna Francyne Silva De Barros.

**Software:** Melania Maria Amorim, Leila Katz.

**Supervision:** Leila Katz.

**Validation:** Melania Maria Amorim, Duana Gabrielle De Lemos Costa, Leila Katz.

**Visualization:** Duana Gabrielle de Lemos Costa, Leila Katz.

**Writing – original draft:** Joanna Francyne Silva De Barros, Duana Gabrielle De Lemos Costa, Leila Katz.

**Writing – review & editing:** Melania Maria Amorim, Leila Katz.
